# Voice as a biomarker: exploratory analysis for benign and malignant vocal fold lesions

**DOI:** 10.3389/fdgth.2025.1609811

**Published:** 2025-08-12

**Authors:** Phillip Jenkins, Rylan Harrison, Steven Bedrick, Lisa Karstens, Bridge2AI-Voice Consortium, Yael Bensoussan, William Hersh

**Affiliations:** ^1^ Division of Informatics, Clinical Epidemiology, Oregon Health and Science University, Portland, OR, United States; ^2^ Fariborz Maseeh Department of Mathematics and Statistics, Portland State University, Portland, OR, United States

**Keywords:** voice biomarkers, Bridge2AI, machine learning (ML), laryngeal lesions, voice 2 AI

## Abstract

Benign and malignant vocal fold lesions can alter voice quality and lead to significant morbidity or, in the case of malignancy, mortality. Early, noninvasive identification of these lesions using voice as a biomarker may improve diagnostic access and outcomes. In this study, we analyzed data from the initial release of the Bridge2AI-Voice dataset to evaluate which acoustic features best distinguish laryngeal cancer and benign vocal fold lesions from other vocal pathologies and healthy voice function. Seven diagnostic cohorts were grouped into two analyses: the first included participants with laryngeal cancer, benign lesions, or no voice disorder; the second included those with laryngeal cancer or benign lesions without other voice disorders, as well as individuals with spasmodic dysphonia or vocal fold paralysis. Acoustic features including fundamental frequency, jitter, shimmer, and harmonic-to-noise ratio (HNR) were extracted from standardized speech recordings and compared using nonparametric statistical methods. Among the overall sample, significant differences were identified in HNR and fundamental frequency between benign lesions and both healthy controls and laryngeal cancer. In cisgender men, these distinctions were also observed, particularly in HNR and its variability. No statistically significant differences were observed among cisgender women, likely due to the limited sample size. These findings suggest that HNR, particularly its variability, may hold promise as a voice-based marker for early detection and monitoring of vocal fold lesions. Further research with larger, more diverse populations is needed to refine these features and validate their clinical utility.

## 1 Introduction

As part of the National Institutes of Health (NIH) Bridge to Artificial Intelligence (Bridge2AI) consortium (1), the Voice to AI project aims to develop voice as a biomarker of health for use in clinical care. The aim is to generate a large, multi-institutional, ethically sourced, and diverse voice database linked to multimodal health biomarkers, thereby fueling voice AI research (1). The early collection of this data was analyzed by students from the inaugural Voice AI Summer School, the first specialized training program in utilizing voice data for the development of AI models (1).

Voice disorders are defined as impairments in the pitch, loudness, or quality of voice that interfere with communication and social participation (2). These disorders may stem from various causes, including vocal fold pathology, neurologic conditions, or functional voice use patterns. Individuals affected by voice disorders often experience reduced quality of life, work-related disability, and social isolation, particularly when vocal communication is central to their professional roles (2, 3). While vocal fold lesions are a common cause of voice disorders, they represent only a subset of the broader etiologic spectrum. One of the conditions of interest was the presence of both benign and malignant vocal fold lesions.

Benign vocal fold lesions can affect human voices and cause morbidity, whereas malignant lesions can cause morbidity and mortality if not treated (2). The prevalence of these conditions is 12.47% for benign lesions (4). There were 13,150 cases of laryngeal cancer reported in 2017, with 3,710 associated deaths (5). One of the first symptoms presented by patients with glottic organic lesions is dysphonia (6). Such complaints require a diagnostic process that includes visualization of the larynx and assessment of the lesion's morphology through video endoscopy (6). Voice, speech, and respiratory sounds provide important clinical insights into patients' health status. In the age of artificial intelligence (AI), patients' audio recordings are being investigated as digital biomarkers for early detection of a broad range of conditions, including laryngeal pathology, neurological and psychological disorders, head and neck cancers, and diabetes (7). The main diseases that affect the vocal folds, leading to lesions, are laryngeal cancer and benign vocal fold lesions (8). Laryngeal cancer is a malignancy arising from the larynx, the anatomical structure in the neck that houses the vocal folds. The vocal folds are paired tissue bands that vibrate as air passes through them, generating sound and enabling speech. Lesions on the vocal folds can impair this vibration, leading to voice changes or loss of phonation (9). Benign Vocal Fold Nodules are non-malignant growths of abnormal tissue on the vocal cords. Common benign lesions of the vocal folds include vocal fold nodules, polyps, cysts, polypoid degeneration, vocal process granulomas, and recurrent respiratory papillomatosis (10). Diagnosis typically involves direct visualization of the vocal folds using a flexible or rigid endoscope inserted through the nose or mouth. Laryngologists or voice-specialized speech-language pathologists perform this outpatient procedure. While biopsy is necessary for definitive diagnosis of malignancy, many benign lesions are diagnosed based on appearance and clinical context. Access to specialized care for laryngeal visualization can be limited outside of major urban centers with interdisciplinary voice clinics (10). The ability to use voice as a biomarker for the early detection and screening of these diseases has far-reaching implications for increasing access to care for underserved populations. It would provide a noninvasive way to screen for these potentially life-changing conditions.

When attempting to detect the presence of vocal lesions, it is essential to determine whether or not the participant has a concordant vocal disorder (11). To use vocal biomarkers specific for vocal fold lesions, understanding other vocal pathologies in the dataset participants must be acknowledged.

The Project Aim is to examine which acoustic features best distinguish laryngeal cancer and benign vocal cord lesions from other vocal pathologies and healthy laryngeal function utilizing the Bridge2AI-Voice v1.1 dataset (12). Acoustic features refer to the measurable properties of the voice signal, including pitch, loudness, and quality. The objective analysis of these features plays a critical role in clinical voice assessments, providing quantifiable data to support diagnosis and treatment planning (13). Beginning with F0, the fundamental frequency is the number of cycles of opening and closing the glottis within a time frame or the frequency at which the vocal cords vibrate. Fundamental frequency conveys pitch and intonation; variation across sex, age groups, and mental states is expected (14).

Closely related is jitter, which is used to measure fluctuations in fundamental frequency. Local jitter is the difference between two consecutive periods (i.e., the length of time to complete one sound wave cycle) divided by the mean period. Higher local jitter percentages correspond to lower control of vocal cord vibration and are regularly found in patients with vocal pathologies (13).

Similarly, shimmer measures fluctuations in the amplitude of sound waves. High shimmer measurements are perceived as breathiness and are correlated with glottal resistance, which can be caused by lesions that interfere with vocal cord movement. For this analysis, we extracted the mean local shimmer, which is the mean difference in consecutive sound wave amplitudes in decibels (dB).

Finally, the harmonic-to-noise ratio (HNR) is the ratio of the periodic to aperiodic component in a speech signal. The periodic component stems from regular glottal pulses during phonation, while the aperiodic component is the noise produced from turbulence as air flows through the glottis. A possible source of this turbulence is the improper closing of the vocal cords (14). We examined both the mean and the standard deviation of the harmonic-to-noise ratio, as we felt the latter would help us measure consistency in vocal production.

The selection of these features was based on the findings of previous related work. For example, Dr. Tom Karlsen and colleagues found that jitter, shimmer, and noise to harmonic ratio were larger among laryngeal cancer patients than among controls using *post hoc* Bonferroni analyses (*P* < 0.001) (15). Likewise, in a study of 112 men with vocal fold leukoplakia, a type of lesion most commonly caused by smoking – Dr. Young Ae Kang and colleagues found higher F0 among those with carcinoma relative to those without using an analysis of covariance (*P* < 0.000) (16).

## 2 Methods

### 2.1 Dataset

The dataset used for this project was the Bridge2AI-Voice v1.0, the initial release, provides 12,523 recordings for 306 participants collected across five sites in North America (9).

### 2.2 Definition of cohorts and groupings

In exploring the potential for a biomarker of vocal cord lesions, we had two related but different clinical objectives. First, we wished to identify acoustic features that could distinguish the voices of participants *with lesions* from those with no vocal pathology at all; and second, we wished to distinguish the voices of participants *with lesions* and from those with *other vocal disorders*. The intersection between participants in our dataset with lesions and those with other vocal disorders [*n* = 6 lesion-present participants who also had either spasmodic dysphonia or unilateral vocal fold paralysis (UVFP)] required breaking down the lesion cohort into participants with lesions and no other vocal disorders for valid comparison against the spasmodic dysphonia and UVFP cohorts. This separation allowed for a sound examination of what acoustic features set apart vocal cord lesions from other vocal pathologies.

**Figure 1:**
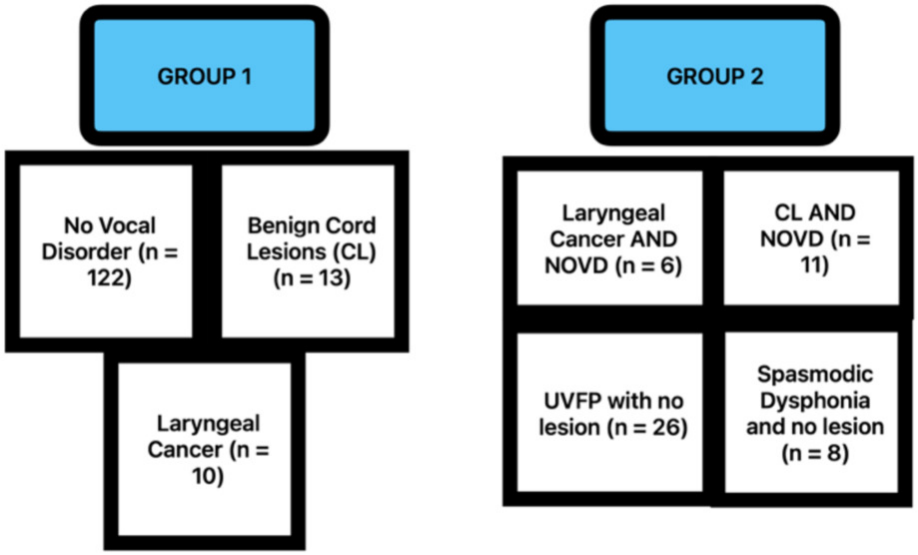
Participant grouping by lesion type and vocal disorder diagnosis.

Since the lesion present with no other voice disorder cohorts were subsets of the lesion present cohorts, thereby introducing statistically dependent cohorts, hypothesis testing was conducted in two groups to ensure the diagnostic cohorts within them contained mutually independent observations. Group 1 consists of recordings for participants with: laryngeal cancer (*n* = 10), benign cord lesions (CL) (*n* = 13), and no voice disorder (NVD) (*n* = 122). Group 2 consists of recordings for participants with: laryngeal cancer with no other voice disorder (NOVD) (*n* = 6), benign CL with NOVD (*n* = 11), spasmodic dysphonia with no lesion (*n* = 8), and UVFP with no lesion (*n* = 26) (Figure 1).

### 2.3 Statistical analysis

Prior to comparing distributions of acoustic features among these cohorts, basic demographic information was analyzed and compared across the lesion-absent and lesion-present cohorts to detect potential biases (Table 1). Continuous variables were compared using the Python library TableOne's (0.9.1) implementation of the Kruskal–Wallis test. Categorical variables were compared using Fisher's exact test using the R Stats package, accessed via a Python environment using rpy2 (3.5.16).

**Table 1:** Demographics and clinical characteristics, grouped by presence of vocal fold lesions.

Characteristic	Overall	Lesion absent	Lesion present	*P*-value
*n*	176	153	23	
Age (years), median	59.0	59.0	60.0	0.260
Weight (lbs), median	169.0	164.0	184.0	0.110
Gender Identity, *n* (%)				
Female	110 (63.2)	97 (64.2)	13 (56.5)	0.546
Male	59 (33.9)	52 (34.4)	7 (30.5)	
Non-binary or genderqueer	2 (1.1)	2 (1.3)	0 (0.0)	
Sexual orientation, *n* (%)				
Bisexual	8 (4.6)	8 (5.3)	0 (0.0)	
Heterosexual	138 (79.3)	119 (78.0)	19 (82.6)	
Homosexual	7 (4.0)	6 (3.9)	1 (4.3)	
Other	5 (2.9)	5 (3.3)		
No answer	16 (9.2)	13 (8.6)	3 (13.0)	
Race, *n* (%)				0.149
American Indian or Alaska Native	1 (0.6)	1 (0.7)		
Asian	7 (4.0)	7 (4.6)		
Black or African American	11 (6.3)	7 (4.6)	4 (17.4)	
White	140 (80.5)	124 (82.1)	16 (69.6)	
Multiracial	6 (3.4)	5 (3.3)	1 (4.3)	
No answer	3 (1.7)	2 (1.3)	1 (4.3)	
Other	6 (3.4)	5 (3.3)	1 (4.3)	
Ethnicity, *n* (%)				0.794
Hispanic or Latino	17 (9.8)	16 (10.6)	1 (4.3)	
Not Hispanic or Latino	148 (85.1)	127 (84.1)	21 (91.3)	
No answer	9 (5.2)	8 (5.3)	1 (4.3)	

Acoustic features were extracted from recordings for the Rainbow Passage task, a paragraph containing all phonemes in American English commonly used as an assessment by speech pathologists. Acoustic features for these recordings were pre-extracted and included in the Bridge2AI dataset by default. They were obtained using openSMILE (17) and stored in PyTorch (18) files. Features for 180 recordings were analyzed across the 176 unique participants with a Rainbow Passage task recording. Four participants out of the 118 represented in the NVD cohort contributed two recordings for this task, while the remaining 172 contributed one. Because those four recordings belonged to the largest cohort, no abnormalities were detected when analyzing their associated acoustic features. Additionally, since there were no objective measures to verify recording quality for each participant, all 180 recordings were used for analysis.

Features examined for analysis were mean HNR, the standard deviation of harmonic-to-noise ratio (HNR SD), mean local jitter, mean local shimmer, and mean fundamental frequency. Analysis was initially conducted collectively for all participants. First, a Kruskal–Wallis test was used to assess differences within Group 1 and then within Group 2 for each acoustic feature. If statistically significant differences were detected (*α* = 0.05), Dunn's test was used to compare all pairs of diagnostic cohorts within each group. *P*-values were adjusted with Holm's method for multiple comparisons. Given the confounding influence of sex on the normal ranges for the selected acoustic features, this analysis was then repeated separately for cisgender men and cisgender women. Transgender individuals were excluded from these stratified analyses because there was no way to verify whether such individuals had received gender-affirming care affecting vocal characteristics.

Statistical tests were conducted in Python (3.10.14) using SciPy (1.13.1) (19) for Kruskal–Wallis tests and Scikit-postdocs (0.9.0) (20) for Dunn's tests.

## 3 Results

Table 1 indicates no statistically significant differences in age, weight, gender identity, sexual orientation, race, or ethnicity between participants with and without a lesion. However, the lesion-present cohort included 12.8% more African Americans than the lesion-absent cohort. In addition, the median weight for the lesion-present cohort was 20 pounds higher than that for the lesion-absent group. Overall, the dataset is predominantly composed of white, heterosexual, and female individuals.

For the analysis representing all 176 participants, statistically significant differences were found between the benign CL and NVD cohorts in their distributions of mean HNR (*p* = 0.019), HNR SD (*p* = 0.028), and fundamental frequency (*p* = 0.012). Additionally, differences were found between benign CL and laryngeal cancer for HNR SD (*p* = 0.028). Results for all Group 1 pairwise comparisons for the unstratified data are shown in Table 2. No statistically significant differences for local jitter and shimmer were found within Group 1, and no statistically significant differences were found within Group 2 for all acoustic features examined.

**Table 2:** Dunn's test results group 1 pairings (unstratified data).

Acoustic feature	Pairing	*p*-value
Mean HNR	Laryngeal cancer, benign C.L.	0.095
Mean HNR	Laryngeal cancer, no voice disorder	0.914
Mean HNR	**Benign C.L., no voice disorder**	**0**.**019**
Standard deviation HNR	**Laryngeal cancer, benign C.L.**	**0**.**028**
Standard deviation HNR	Laryngeal cancer, no voice disorder	0.256
Standard deviation HNR	**Benign C.L., no voice disorder**	**0**.**028**
Mean F0	Laryngeal cancer, benign C.L.	0.335
Mean F0	Laryngeal cancer, no voice disorder	0.429
Mean F0	**Benign C.L., no voice disorder**	**0**.**012**

Bolded values indicate statistical significance at the *p* < 0.05 level.

The number of recordings for each diagnostic cohort, stratified by diagnostic cohort, is shown in Table 3. The analysis, which consisted only of cisgender men (Table 4), revealed statistically significant differences between the benign CL and NVD cohorts for mean HNR (*p* = 0.004) and HNR standard deviation (*p* = 0.002). Moreover, differences were once again detected between benign CL and laryngeal cancer in their respective distributions of HNR SD (*p* = 0.027). The initial Kruskal–Wallis test indicated statistically significant differences within Group 2 for HNR SD (*p* = 0.03), but this was not supported by the *post-hoc* Dunn's test; the smallest adjusted *p*-value was 0.055, produced from the laryngeal cancer NOVD and benign CL NOVD comparison. Differences were not detected among distributions for any other features.

**Table 3:** Number of recordings for cisgender men and women, by diagnostic cohort.

Diagnostic group	# Cisgender women recordings	# Cisgender men recordings
Laryngeal cancer	6	4
Benign CL	7	6
No voice disorder	77	36
Laryngeal cancer (NOVD)	2	4
Benign CL (NOVD)	6	5
Spasmodic Dysphonia + no lesion	6	2
UVFP + no lesion	17	9

**Table 4:** Dunn's test results for group 1 pairings with only cisgender male participants.

Acoustic feature	Pairing	*p*-value
Mean HNR	Laryngeal cancer, benign C.L.	0.192
Mean HNR	Laryngeal cancer, no voice disorder	0.512
Mean HNR	**Benign C.L., no voice disorder**	**0**.**004**
Standard deviation HNR	**Laryngeal cancer, benign C.L.**	**0**.**027**
Standard deviation HNR	Laryngeal cancer, no voice disorder	0.863
Standard deviation HNR	**Benign C.L., no voice disorder**	**0**.**002**

Bolded values indicate statistical significance at the *p* < 0.05 level.

No statistically significant differences were found among cisgender women for all acoustic features examined.

## 4 Discussion

Our preliminary analysis of the Bridge2AI-Voice dataset shows early promise that there are vocal features that can act as a biomarker for vocal fold lesions. Other recent studies have shown links between benign and malignant vocal fold lesions using principal component analysis (PCA), suggesting the utility of the PCA method in the identification of vibrational alterations in the acoustic characteristics of voice affected by lesions (21). Interestingly, Liu et al.'s PCA analysis highlighted an underlying acoustic difference between multiple conditions, such as Reinke's edema, polyps, cysts, and leukoplakia (21).

Despite the relatively small sample size, we detected statistically significant differences in acoustic features within our Group 1 cohort. Notably, the differences were most pronounced between the benign C.L. cohort and the NVD cohort.

Of particular interest is the difference in HNR SD between benign and malignant lesion groups, which suggests that HNR SD may be a useful measure for monitoring lesion progression and detecting laryngeal cancer at an early stage. This is a finding that will be interesting to test with larger datasets, and future studies can potentially leverage this to explain this relationship further. However, no statistically meaningful differences were found within Group 2, indicating that distinguishing lesions from other vocal pathologies may be more challenging.

The primary limitations of this study were the small sample size and participants' incomplete lesion histories. Despite these limitations, the study provides valuable insights into the potential for voice biomarkers to serve as early indicators of vocal fold lesions.

The most striking barrier for our selected features to be considered for a biomarker of vocal cord lesions is that, when we stratified our data by sex, we found no statistically significant differences among women for Groups 1 or 2. The power of these statistical tests was, of course, limited by the small sample sizes in some of these cohorts, most noticeably when comparing against the 2 cisgender women participants in the laryngeal cancer + no other vocal disorder cohort, as shown in Table 4. Even so, the fact that no differences were detected among either group for cisgender women suggests we should broaden our search to additional acoustic features. For cisgender men, differences were only found when comparing distributions for mean and SD HNR. Differences were found among benign CL and no voice disorder for both as well as between benign CL and laryngeal cancer for SD HNR, which aligns with the results for the unstratified data. Another notable finding is that even though the results of the Kruskal–Wallis test indicated significance differences within group 2 for cisgender men when comparing SD HNR, the *post-hoc* analysis did not back that up, though we did approach significance for the benign C.L. (NOVD) + laryngeal cancer (NOVD) comparison (*p* = 0.055).

Additionally, voice disorders arising from a broader range of laryngeal diseases, such as spasmodic dysphonia, vocal fold paralysis, and functional dysphonia, carry significant morbidity and impair communication and quality of life (22). Recent advances in artificial intelligence have enabled voice recordings to distinguish between different laryngeal pathologies with increasing accuracy. Studies have shown that convolutional neural networks and deep learning models trained on spectrogram representations can classify laryngeal diseases, including early laryngeal cancer, with promising results using standard microphone recordings or even smartphone-captured voice samples (23). These approaches offer a noninvasive, scalable, and accessible method to augment current diagnostic workflows and may serve as effective screening tools for laryngeal malignancy in primary care and underserved settings. As AI protocols mature and datasets grow more diverse, their integration into clinical voice screening may become an important complement to traditional laryngoscopy.

While a definitive diagnosis still requires visualization, a validated AI-based voice screening tool could serve as a triage mechanism. It could identify individuals with subtle voice changes who may not otherwise seek care, especially in primary care or telehealth settings. Such a tool could prompt earlier referrals to voice specialists, help prioritize urgent cases, and reduce diagnostic delays. Unlike the human ear, which may not reliably distinguish between subtle pathologic changes, an AI model can offer consistent and scalable voice analysis across diverse populations.

Future studies should focus on increasing sample sizes and incorporating more nuanced data, such as lesion sizes. Additionally, the sex of participants played a role in the results, which should be considered in future recruitment efforts to prevent biased datasets. Further research should continue to explore different types of benign and malignant lesions by voice feature.

## Data Availability

The original contributions presented in the study are included in the article/Supplementary Material, further inquiries can be directed to the corresponding author.

## Group members of Bridge2AI-Voice Consortium

University of South Florida, Tampa, FL, US: Yael Bensoussan. Weill Cornell Medicine, New York, NY, USA: Olivier Elemento. Weill Cornell Medicine, New York, NY, USA: Anais Rameau. Weill Cornell Medicine, New York, NY, USA: Alexandros Sigaras. Massachusetts Institute of Technology, Boston, MA, USA: Satrajit Ghosh. Vanderbilt University Medical Center, Nashville, TN, USA: Maria Powell. University of Montreal, Montreal, Quebec, Canada: Vardit Ravitsky. Simon Fraser University, Burnaby, BC, Canada: Jean Christophe Belisle-Pipon. Oregon Health & Science University, Portland, OR, USA: David Dorr. Washington University in St. Louis, St. Louis, MO, USA: Phillip Payne. University of Toronto, Toronto, Ontario, Canada: Alistair Johnson. University of South Florida, Tampa, FL, USA: Ruth Bahr. University of Florida, Gainesville, FL, USA: Donald Bolser. Dalhousie University, Toronto, ON, Canada: Frank Rudzicz. Mount Sinai Hospital, Sinai Health, University of Toronto, Toronto, ON, Canada: Jordan Lerner-Ellis. Boston Children's Hospital, Boston, MA, USA: Kathy Jenkins. University of Central Florida, Orlando, FL, USA: Shaheen Awan. University of South Florida, Tampa, FL, USA: Micah Boyer. Oregon Health & Science University, Portland, OR, USA: William Hersh. Washington University in St. Louis, St. Louis, MO, USA: Andrea Krussel. Oregon Health & Science University, Portland, OR, USA: Steven Bedrick. UT Health, Houston, TX, USA: Toufeeq Ahmed Syed. University of South Florida, Tampa, FL, USA: Jamie Toghranegar. University of South Florida, Tampa, FL, USA: James Anibal. New York, NY, USA: Duncan Sutherland. University of South Florida, Tampa, FL, USA: Enrique Diaz-Ocampo. University of South Florida, Tampa, FL, USA: Elizabeth Silberhoz Boston Children's Hospital, Boston, MA, USA: John Costello. Vanderbilt University Medical Center, Nashville, TN, USA: Alexander Gelbard. Vanderbilt University Medical Center, Nashville, TN, USA: Kimberly Vinson. University of South Florida, Tampa, FL, USA: Tempestt Neal. Mount Sinai Health, Toronto, ON, Canada: Lochana Jayachandran. The Hospital for Sick Children, Toronto, ON, Canada: Evan Ng. Mount Sinai Health, Toronto, ON, Canada: Selina Casalino. University of South Florida, Tampa, FL, USA: Yassmeen Abdel-Aty. University of South Florida, Tampa, FL, USA: Karim Hanna. University of South Florida, Tampa, FL, USA: Theresa Zesiewicz. Florida Atlantic University, Boca Raton, FL, USA: Elijah Moothedan. University of South Florida, Tampa, FL, USA: Emily Evangelista. Vanderbilt University Medical Center, Nashville, TN, USA: Samantha Salvi Cruz. Weill Cornell Medicine, New York, NY, USA: Robin Zhao. University of South Florida, Tampa, FL, USA: Mohamed Ebraheem. University of South Florida, Tampa, FL, USA: Karlee Newberry. University of South Florida, Tampa, FL, USA: Iris De Santiago. University of South Florida, Tampa, FL, USA: Ellie Eiseman. University of South Florida, Tampa, FL, USA: JM Rahman. Boston Children's Hospital, Boston, MA, USA: Stacy Jo. Hospital for Sick Children, Toronto, ON, Canada: Anna Goldenberg.
