# Supplementary figures and images for: Voice as a biomarker: exploratory analysis for benign and malignant vocal fold lesions
Source: Front Digit Health. 2025 Aug 12;7:1609811. doi: 10.3389/fdgth.2025.1609811 (PMC12378753; doi:10.3389/fdgth.2025.1609811)

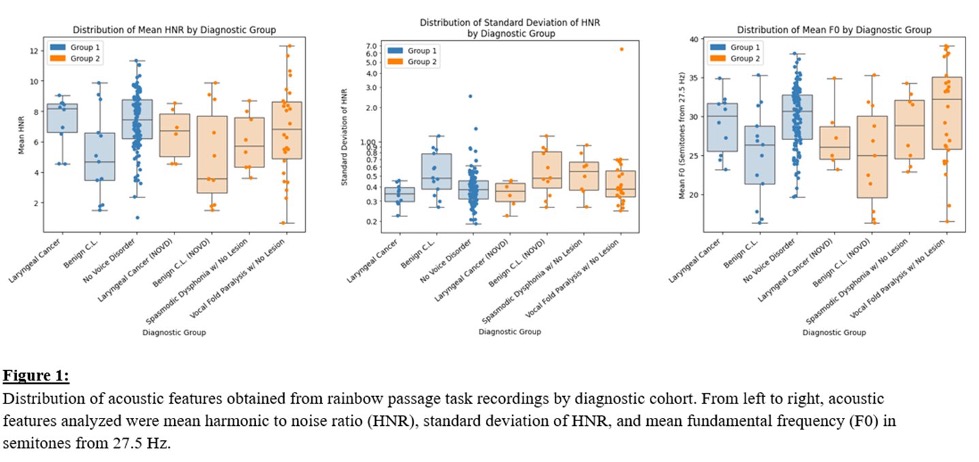

Supplement: Supplementary file 1 [file Image1.jpeg]
